# Malaria morbidity, mortality and associated costs in Indonesia: analysis of the National Health Insurance claim dataset

**DOI:** 10.1136/bmjgh-2024-018255

**Published:** 2025-05-12

**Authors:** Ery Setiawan, Angela Devine, Helen Dewi Prameswary, J Kevin Baird, Ric Price, Kamala Thriemer

**Affiliations:** 1 Division of Global and Tropical Health, Menzies School of Health Research, Charles Darwin University, Darwin, Northern Territory, Australia; 2 Health Administration and Policy, Universitas Indonesia, Depok, Indonesia; 3 Centre for Epidemiology and Biostatistics, Melbourne School of Population and Global Health & Centre for Health Policy, University of Melbourne, Melbourne, Victoria, Australia; 4 National Malaria Program, Ministry of Health of the Republic of Indonesia, Jakarta, Indonesia; 5 Clinical Research Unit, Oxford University, Central Jakarta, Indonesia; 6 Nuffield Department of Medicine, University of Oxford, Oxford, UK

**Keywords:** Malaria, Health insurance, Health economics

## Abstract

**Introduction:**

Data on morbidity, mortality and cost for malaria-related hospitalisation are important for prioritising resources for malaria control strategies, but these data are often limited. The aim of this study was to understand the current malaria service delivery in Indonesia, including referral rates to hospitals, mortality outcomes and malaria-related costs at hospitals, using data from National Health Insurance claims.

**Methods:**

Data were gathered from the recent Indonesian National Health Insurance dataset for claims made between 2015 and 2020. Cases were selected for any diagnosis with the international classification of diseases-10th revision codes for malaria-related diseases. Patients’ sociodemographic status, repeated presentations to healthcare facilities, referral patterns and costs of treatment for the hospital settings were assessed by malaria species. Costs were reported in 2020 US$.

**Results:**

Data were available for 12 970 episodes of malaria, which occurred in 8833 patients. *Plasmodium falciparum* accounted for 6019 (46.4%) episodes, and *P. vivax* for 4307 (33.2%) episodes. The incidence rates were 0.38 (95% CI 0.29 to 0.47) per person-years for *P. falciparum* and 0.33 (95% CI 0.19 to 0.52) for *P. vivax*. 46% of malaria cases initially presented at the hospital. Among these patients, the mean cost was US$16.2 (SD 4.4) for an outpatient consultation and US$228.7 (SD 122.6) for inpatient care. In total, 4.8% (623) of patients re-presented to the hospital within 30 days of a malaria episode, of whom 1.7% (219) required admission for inpatient care, which was estimated to cost US$230.0 (SD 105.5). The risk of mortality for inpatients with *P. falciparum* malaria was 2.1% (36/1718) compared with 1.2% (16/1359) for patients with *P. vivax* malaria; p=0.069.

**Conclusions:**

The National Health Insurance claim data provide detailed costing estimates. Integrating data from the existing malaria information system with the data from the National Health Insurance claims can provide important insights into the healthcare costs associated with the management of malaria that could help optimise national antimalarial policy.

**WHAT IS ALREADY KNOWN ON THIS TOPIC**

- The routine malaria surveillance system in Indonesia collects data on morbidity, mortality and treatment; but it is missing important information relevant for decision-making, including comorbidities, care pathways and associated healthcare costs. These additional data, however, are captured in the National Health Insurance claim dataset.

**WHAT THIS STUDY ADDS**

- More than half of the malaria cases were treated at primary care facilities. While the majority of patients requiring inpatient care were considered low severity, malaria mortality rates were higher than that estimated by the Ministry of Health. This study is the first to report cost data from the Indonesian National Health Insurance dataset, providing detailed estimates for patients presenting with different malaria species at the hospital level.

**HOW THIS STUDY MIGHT AFFECT RESEARCH, PRACTICE OR POLICY**

- Integrating the National Health Insurance dataset into routine surveillance data could provide more granular data for the Ministry of Health to direct malaria control and elimination efforts. Future studies could also elaborate on the potential of linking other disease information systems with the National Health Insurance data.

## Introduction

 Malaria is a global health priority identified under Sustainable Development Goal 3.3, which aims to end epidemics, including malaria, by 2030.^1^ Estimates from 2022 suggest 249 million malaria cases occurred in 85 malaria-endemic countries, and there have been no improvements in reducing the global burden of malaria since 2015.^2^

In Indonesia, malaria is a health sector priority as mandated in the National Mid-term Development Plan 2020–2024.^3^ A total of 372 districts in Indonesia were certified as malaria-free in 2022, while the remaining 142 districts are categorised as low- to high-endemicity areas.^4^ Similar to the global trend,^2^ the overall number of cases in Indonesia has increased from 254 055 in 2020 and 304 607 in 2021 to more than 400 000 in 2022.^4^ The greatest burden in Indonesia is in the eastern provinces of the archipelago, particularly Papua and West Papua. Indonesian malaria surveillance reports showed a gradual decrease in malaria cases in non-Papua provinces between 2011 and 2022, while there was an increase in Papua in the same time period.^4^

The control and elimination of malaria in Indonesia is confounded by many contributing factors, including limited access to care and treatment-seeking behaviour in remote and poorly resourced locations. A study from Central Sulawesi, East Nusa Tenggara, Maluku, Papua and West Papua Provinces showed that people who were unsure when to seek care and had limited awareness of health facilities, including not knowing where the nearest health facility was, were more than fourfold more likely to have a history of malaria.^5^ A recent systematic review highlighted that Indonesian patients with malaria had a huge spectrum of treatment-seeking behaviour that ranged from doing nothing to self-treatment (purchasing medicine from the drug store) to actively seeking treatment at health facilities.^6^ The differences in health-seeking behaviours were attributed to education, socioeconomic level and geographical issues in relation to access to and perception of malaria treatment.^6^

Malaria illness affects the individual patient but also impacts economic costs incurred by households and the health system. The economic burden on households includes productivity losses due to illness or caretaking of the patient and any out-of-pocket expenditures related to managing the symptoms prior to visiting the healthcare facility and seeking care outside of the public system. The economic impact on the health system includes costs for resources such as drugs and equipment as well as human resources.

There are limited data available on the economic burden of malaria in general and by malaria species on patients and the healthcare system in Indonesia. The inpatient cost for patients presenting with severe falciparum malaria in 2016 was estimated to be between US$55 and US$66, depending on the treatment.^7^ Provider cost data from studies focusing on vivax malaria estimated those in the primary care setting at US$6.6.^8^ To our knowledge, no detailed data on the cost of hospitalisation by species and/or severity of disease or the costs related to comorbidity and re-presentation are available.

The Indonesian National Health Insurance (NHI) claim dataset has the potential to provide insights into the costs of malaria treatment and care as it records healthcare utilisation for individual patients. The NHI scheme currently covers around 95% of the entire population, and it includes coverage for malaria treatment.^9^ The open-access NHI claims data are produced annually from a weighted cross-sectional sample, including data at primary care facilities and hospitals, to represent the 1% of healthcare utilisation under the NHI scheme.^10^ The claims dataset includes the sociodemographic status of the individual patient, their healthcare visits, including dates, referral and discharge status in healthcare facilities, as well as the costs associated with hospital visits.^10^ The aim of this study was to use the NHI claim datasets to understand the current cascade of care for malaria service delivery in Indonesia, including referral rates to hospitals, mortality outcomes and malaria-related costs.

## Methods

### Study design and data source

This analysis used data from the most recently published NHI claim dataset between 2015 and 2020, which included data from all 34 provinces in Indonesia.

Four NHI attribute datasets were included in this analysis: (1) membership dataset, (2) primary-care dataset, (3) hospital dataset and (4) secondary diagnosis dataset.^10^ The data were derived using stratified random sampling by considering household categories, including those who never received services, those who received services at primary care and those who received services at primary care and at the hospital. Household-level weight scores were generated to reflect how well the sampled data represented the actual population.

In this dataset, all interactions with the healthcare system can be traced for the cohort of sampled individuals, including healthcare visits not specific to malaria. Services in all public and registered private-owned facilities are recorded, but medication details, such as the drug’s name and dosage, are not available.

The *membership dataset* includes sociodemographic information such as gender, date of birth, type of membership and residency. Type of membership indicates those whose premiums were paid by the government (poor and near poor population as registered by the Ministry of Social Affairs) and those who paid the premium themselves (paid and unpaid workers). The first type of membership (paid by the Government) is entitled to the third class of inpatient ward, while the paid workers are eligible in the second or first class. The individual unpaid workers are entitled to either first, second or third class depending on the level of contribution (premium) rate. The membership types incur different costs for inpatient care at the hospital level based on differently set tariffs of the inpatient ward classes.^11^

The *primary care and hospital datasets* consist of detailed clinical information, including the primary diagnosis using the international classification of diseases-10th revision (ICD-10), referral condition, type of facility where the service was provided, whether the service was conducted during an outpatient or inpatient visit, discharge status and care-associated cost per visit.

The *primary care dataset* covers service records of three types of facilities, including Puskesmas (government-owned primary care facilities), private general practitioner (GP) and private clinics that usually consist of more than one GP and/or specialist.^12^ Hospital visit records often include more than one diagnosis, especially for inpatient stays; but the *hospital dataset* only covers the primary diagnosis. If more than one diagnosis is recorded, these are captured in the *secondary diagnosis dataset* (online supplemental table 1).

### Data management

All four datasets were linked through individual and unique patient IDs. Records of re-presentation care were identified through the unique patients ID and date of visit.

The hospital and secondary diagnosis datasets were combined, and the malaria episodes selected. Patients with malaria who attended a hospital were recorded in the dataset with malaria either as their primary, secondary or tertiary diagnoses. Case selection was made for any diagnosis with the ICD-10 codes for malaria-related disease categories. These ICD-10 codes include B50 (*Plasmodium falciparum*), B51 (*P. vivax*), B52 (*P. malariae*), B53 (other specified malaria, which includes *P. knowlesi* and *P. ovale*) and B54 (unspecified malaria, which includes clinical diagnosis without laboratory confirmation). In Indonesia, malaria diagnoses are usually confirmed by a rapid diagnostic test (RDT). The most commonly used RDT in Indonesia identifies infections caused by *P. falciparum* (using HRP2 antigen capture, which is specific to this species) or so-called non-falciparum infections (using plasmodial LDH antigen capture, which occurs in all species). However, the type of diagnostic used is not recorded in the dataset.^13^ For the purpose of the analysis, results are presented by species for *P. falciparum* (ICD-10 code B50) and *P. vivax* (ICD-10 code B51) separately, as a combined category ‘other malaria’, including *P. malariae*, *P. knowlesi* and *P. ovale* (ICD-10 codes B52 and B53), and unspecified malaria (ICD-10 code B54).

A similar case selection process using the ICD-10 code was also undertaken for cases recorded at the primary care level.

### Data analysis

The patient’s age at the time of presentation to a health facility was calculated from the date of birth and the date of admission.^14^ Socioeconomic status was derived using the membership classification by the premium amount. One episode of inpatient care was defined as the time between the day of admission until the day of discharge. Disease severity for each presentation was defined using the diagnostic-related group code in the hospital dataset with three levels of severity, including low, medium and high severity.^15^

The cascade of services was displayed in a flow chart to highlight the proportion of patients moving through the system from the primary care facility to the hospital until discharge or death. χ^2^ and analysis of variance tests were used to compare proportions as appropriate with the p value as a reference.^16^ The p value is the probability that the observed effect within the study would have occurred by chance and if, in reality, there was no true effect. A p value <0.05 was considered statistically significant.^17^

The incidence of malaria across multiple episodes was expressed per person-years of observation. The observation period was defined as the time between the first episode of malaria until 31 December 2020.

Healthcare costs were presented as means and SD. In patients presenting to the hospital, the costs of inpatient and outpatient care were calculated separately. Since data included a unique identification of each patient, the additional cost of healthcare utilisation due to patients presenting at the hospital within 30 days of an acute malaria episode could be collated.

Inpatient episodes included costs related to comorbidity and associated complications. Costs per malaria episode managed at a hospital were stratified by the duration of stay and the severity of the disease. Cost estimates were derived in local currency (Indonesian Rupiah) and converted into US$ using the exchange rate from 2020 to correspond with the final year of the data.^18^ Inflation was not applied as there were no changes in healthcare tariffs during the observation period in accordance with the Ministry of Health regulations.

### Patient and public involvement

Our study did not involve direct interaction with human respondents, but rather analysed secondary data. All data are publicly available by request to the NHI, providing that the researcher signs an integrity statement to express that data will only be used for research purposes. Ministry of Health personnel are co-authors on this study and were included in conceptualising the study, reviewing the analysis and interpreting the findings.

## Results

During the 6-year study period, a total of 1 762 637 disease episodes were recorded in the NHI dataset, of which 12 970 (0.7%) were malaria episodes, occurring in 8833 patients. A total of 6019 (46.4%) malaria episodes were caused by *P. falciparum*, and 4307 (33.2%) by *P. vivax* (online supplemental table 2).

More than half of those episodes (57.8%; 7498/12 970) occurred in male patients, and this proportion was similar for both *P. falciparum* and *P. vivax* (table 1). The median age of patients with malaria was 29 (IQR: 14–41), with 7.4% (963/12 970) of the malaria episodes occurring in children under 5 years. A total of 5894 (45.4%) malaria episodes occurred in patients in the lowest socioeconomic category (table 1). Overall, 7851 (60.5%) of malaria episodes were reported from three provinces: 5561 (42.9%) from Papua, 1199 (9.2%) from East Nusa Tenggara and 1091 (8.4%) from West Papua (online supplemental table 3). The proportion of malaria due to each species varied between provinces. In Papua, 61.0% (3394/5561) of cases were due to *P. falciparum,* whereas in East Nusa Tenggara, 46% (551/1199) were due to *P. vivax* (online supplemental table 3).

**Table 1:** Malaria episodes in the National Health Insurance claim dataset between 2015 and 2020

	*P. falciparum*		*P. vivax*		Other malaria		Unspecified malaria		Total	
	n	%	n	%	n	%	n	%	N	%
Total episodes	6019	46.4	4307	33.2	260	2.0	2384	18.38	12 970	100
Gender										
Male	3410	56.7	2508	58.2	162	62.3	1418	59.5	7498	57.8
Female	2609	43.3	1799	41.8	98	37.7	966	40.5	5472	42.2
Age group										
0–11 months	46	0.8	53	1.2	2	0.8	10	0.4	111	0.9
1–5 years	369	6.1	343	8.0	6	2.3	134	5.6	852	6.6
6–10 years	594	9.9	488	11.3	26	10.0	201	8.4	1182	9.1
11–15 years	617	10.3	334	7.8	35	13.5	196	8.2	9131	70.4
16–65 years	4206	69.9	2967	68.9	185	71.2	1773	74.4	1309	10.1
Above 65	187	3.1	122	2.8	6	2.3	70	2.9	385	3.0
Membership segment*										
Class I	1223	20.3	1064	24.7	49	18.8	414	17.4	2750	21.2
Class II	1721	28.6	1649	38.3	59	22.7	897	37.6	4326	33.4
Class III	3075	51.1	1594	37.0	152	58.5	1072	45.0	5894	45.4

* Membership in the National Health Insurance claim dataset is categorised by premium amount which is used to define socioeconomic status. Class III is defined as the lowest socioeconomic status with a lower premium rate.

Of the 12 970 malaria episodes, 7006 (54.0%) were recorded at primary healthcare facilities, and 5964 (46.0%) at the hospital level, either as outpatient visits (2526, 42.4%) or inpatient admissions (3438, 57.6%; table 2). Of the 3438 episodes requiring inpatient care, 1718 (50.0%) were due to *P. falciparum,* and 1359 (39.5%) were due to *P. vivax*. The proportion of patients attending primary healthcare facilities or hospitals as inpatient or outpatients was not different between *P. falciparum* and *P. vivax* episodes (p=0.112). The predominant primary care facilities were at the Puskesmas level (government-owned primary care facility), which managed 44.2% (3096/7006) of episodes (table 2).

**Table 2:** Malaria species and type of care received for initial visit to healthcare facilities

	*P. falciparum*		*P. vivax*		Other malaria		Unspecified malaria		Total	
	n	%	n	%	n	%	n	%	N	%
Total episodes	6019	46.4	4307	33.2	260	2.0	2.384	18.4	12 970	100
Primary healthcare	2919	48.5	2157	50.1	190	73.1	1740	73.0	7006	54.0
Private general practitioner	842	28.8	804	37.3	44	23.2	482	27.7	2172	31.0
Private clinics	608	20.8	603	28.0	26	13.7	501	28.8	1738	24.8
Puskesmas[Table-fn T2_FN1]	1469	50.3	750	34.8	120	63.2	757	43.5	3096	44.2
Hospital presentation	3100	51.9	2150	36.1	70	26.9	644	27.0	5964	46.0
Inpatient stays	1718	55.4	1359	63.2	48	68.6	313	48.6	3438	57.6
Presented directly without referral	199	11.6	177	13.0	3	6.3	39	12.5	418	12.2
Primary care referral	869	50.6	714	52.5	21	43.8	149	47.6	1753	51.0
Hospital (horizontal referral)	650	37.8	468	34.4	24	50.0	125	39.9	1267	36.9
Outpatient visits	1382	44.6	791	36.8	22	31.4	331	51.4	2526	42.4
Presented directly without referral	20	1.4	10	1.3	1	4.5	6	1.8	37	1.5
Primary care referral	1160	83.9	684	86.5	12	54.5	227	68.6	2083	82.5
Hospital (horizontal referral)	202	14.6	97	12.3	9	40.9	98	29.6	406	16.1

* Puskesmas are primary healthcare facilities as mandated by the Indonesian government.

### Repeated malaria presentations

Over the 6-year study period, 6676 (75.6%) of the 8833 patients had one recorded episode of malaria, 1330 (15.1%) had two episodes and 827 (9.4%) had more than two episodes (online supplemental table 4). Following an initial presentation with malaria, the incidence rate of *P. vivax* was 0.33 (95% CI 0.21 to 0.46) episodes per person-years compared with 0.38 (95% CI 0.30 to 0.45) per person-years for *P. falciparum* (online supplemental table 5). The incidence rate for both species of malaria did not differ significantly between different age groups, p=0.242.

### Comorbidity

Of the 5964 episodes recorded at a hospital, 2722 (45.6%) were related only to a diagnosis of malaria, 1837 (30.8%) episodes had one additional non-malaria diagnosis and 893 (14.9%) had two additional diagnoses recorded (online supplemental table 6). The additional diagnoses were either due to comorbidity or because of complications related to the malaria diagnosis, such as pneumonia or gastroenteritis.

Of the 12 970 malaria episodes, 4.8% (623) episodes resulted in patients presenting again within 30 days for additional healthcare (online supplemental table 6). Of these, 3.1% (404) were treated as outpatients and 1.7% (219) required admission for inpatient care. Of those requiring inpatient care at re-presentation, 77.2% (169/219) were due to recurrent malaria and 22.8% (50/219) were due to non-malaria illness. Compared with inpatient care, fewer of those re-presenting for outpatient care were due to recurrent malaria (47.5%, 192/404). Patients initially presenting with *P. falciparum* were more likely to present again to the hospital for outpatient care within 30 days (3.5%, 213/6019) compared with those initially presenting with *P. vivax* (2.4%, 102/4307); OR=1.51 (95% CI 1.19 to 1.92), p<0.0001 (online supplemental table 7). No difference was seen between *P. falciparum* and *P. vivax* for inpatient re-presentations.

### Severity

**Figure 1:**
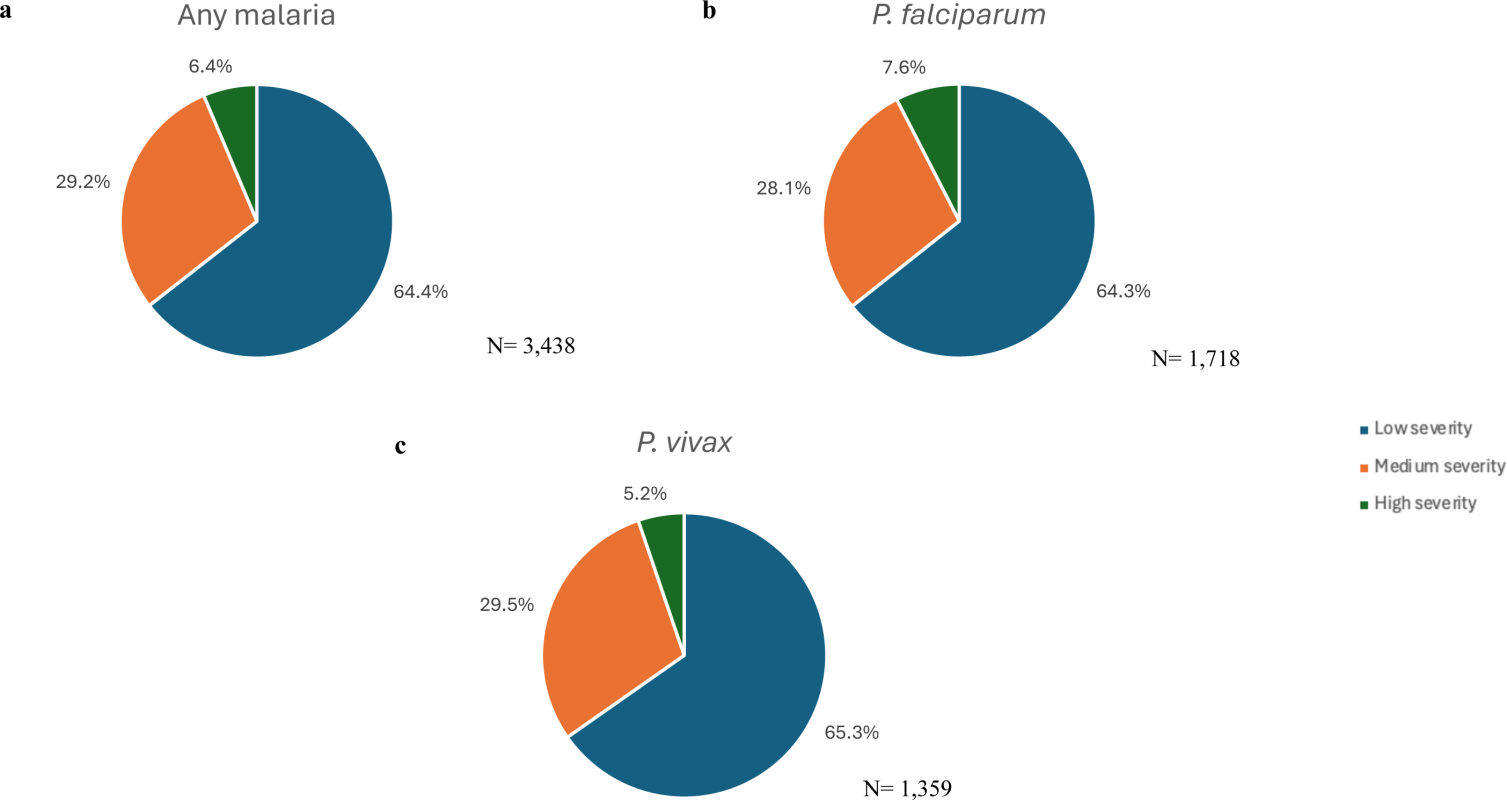
The degree of severity of malaria inpatient cases as classified in the diagnostic-related group code for (**a**) any malaria, (**b**) *P. falciparum*, (**c**) *P. vivax* and (**d**) for other malaria.

In total, 2215 (64.4%) malaria episodes among inpatients were categorised as low severity (level 1), 1004 (29.2%) as medium severity (level 2) and 219 (6.4%) as high severity (level 3) (figure 1). *P. falciparum* episodes were more likely to be classified as high severity level (7.6%, 130/1718), compared with *P. vivax* episodes (5.2%, 71/1359); OR=1.45 (95% CI 1.1 to 1.9), p=0.015.

For the 3438 episodes of malaria that required admission to hospital, the mean duration of stay was 3.9 days (SD 3.2), and this varied from 3.1 days (SD 1.9) for episodes classified as low severity to 5.1 days (SD 4.1) for those of medium severity, and 6.2 days (SD 5.4) for episodes with high severity (table 3). Patients aged 0–11 months and above 65 years old stayed in hospital longer for inpatient stays as compared with other patients (online supplemental table 8). The mean duration of hospital stay was 3.9 days (SD 3.1) for patients with *P. vivax* and 3.7 days (SD 3.0) for those with *P. falciparum*; p=0.379 (online supplemental table 8).

**Table 3:** The mean and SD of length of stay for patients with malaria who are treated in hospital as inpatient, by malaria species and severity level (N=3438)

	*P. falciparum* (n=1718)		*P. vivax* (n=1359)		Other malaria (n=48)		Unspecified malaria (n=313)		Total (N=3438)	
	Mean	SD	Mean	SD	Mean	SD	Mean	SD	Mean	SD
Low severity	2.9	1.7	3.1	2.0	3.5	1.5	3.9	2.2	3.1	1.9
Medium severity	5.0	3.6	4.9	3.9	4.9	2.9	6.2	6.3	5.1	4.1
High severity	5.9	5.7	6.5	5.1	13	N/A	6.1	3.6	6.2	5.4
Overall	3.7	3.0	3.9	3.1	4.3	2.6	4.8	4.2	3.9	3.2

SD, Standard Deviation.

A total of 54 (1.6%) patients admitted to hospital with malaria died during their stay. The mortality rate was 2.1% (36/1718) for patients with *P. falciparum* malaria and 1.2% (16/1359) for patients with *P. vivax* malaria; OR=1.80 (95%CI: 1.02 to 3.33), p=0.069.

### Malaria-related referrals

Overall, 3.0% (207/7006) of patients attending a primary care facility were referred to hospital (online supplemental figure 1 and online supplemental table 9), and this was significantly higher for *P. falciparum* episodes (3.1%, 91/2919) compared with *P. vivax* episodes (1.5%, 33/2157); OR=2.07 (95%CI: 1.40 to 3.13), p=0.0003. In addition, 94% (3231/3438) of patients with a malaria episode requiring inpatient care were discharged as cured, with only 0.1% (36/3438) referred to another hospital (online supplemental table 9). Of the 3438 inpatient episodes, 1753 (51.0%) were referred from the primary care level, 1267 (36.8%) were referred from another hospital and 418 (12.2%) were referred directly (online supplemental figure 1). Referral from another hospital usually occurred from a lower level to a higher level due to the need for more complex equipment or specialists.

### Healthcare costs in patients attending a hospital

Based on the 5964 episodes of malaria management at a hospital, the mean cost for outpatient care was US$16.2 (SD 4.4) and US$228.7 (SD 122.6) for inpatient care (table 4). The costs of care were similar for patients admitted with *P. falciparum*, *P. vivax* and other malaria. Costs increased with each additional non-malaria diagnosis. The cost of patients with a diagnosis of malaria alone was US$192.8 (SD 34.6) per episode, compared with US$229.9 (SD 82.5) for patients with one additional non-malaria diagnosis, US$250.2 (SD 103.8) for two additional diagnoses and US$315.2 (SD 278.2) for more than two additional diagnoses (table 4).

**Table 4:** Malaria cost overall and by species and type of service at hospital in 2020 US$ based on 5964 malaria episodes at hospital level

	*P. falciparum*		*P. vivax*		Other malaria		Unspecified malaria		Total	
	Mean	SD	Mean	SD	Mean	SD	Mean	SD	Mean	SD
Outpatient	16.6	4.2	15.9	4.5	16.5	16.5	16.5	16.5	16.2	4.4
Inpatient	225.6	129.5	231.9	119.1	202.1	51.1	236.6	104.2	228.7	122.6
By number of additional non-malaria diagnoses										
Only malaria diagnosis	190.7	34.5	195.4	33.3	218.4	41.6	236.1	86.3	192.8	34.6
Malaria and one additional diagnoses	226.5	85.2	232.9	79.3	238.5	75.3	266.2	103.7	229.9	82.5
Malaria and two additional diagnoses	252.0	118.5	244.9	84.3	181.0	76.7	374.2	190.4	250.2	103.8
Malaria and >2 additional diagnoses	302.9	291.6	327.4	278.3	190.6	31.2	193.8	39.2	315.2	278.2
By severity level										
Low severity	191.2	34.7	197.6	33.9	191.4	32.7	196.5	39.1	194.2	34.8
Medium severity	263.6	105.9	276.5	100.9	221.9	73.3	284.3	121.1	270.2	105.5
High severity	376.2	364.5	407.4	380.8	214.7	N/A	397.2	193.8	387.2	358.2
By length of stay										
1 day	187.9	43.9	235.3	209.0	239.5	75.1	183.8	37.9	205.8	131.5
2 days	204.9	62.1	206.0	46.2	196.4	29.3	226.4	114.2	206.4	60.5
3 days	217.2	90.8	215.7	57.1	212.0	38.8	211.8	58.5	216.1	75.0
>3 days	256.5	185.8	258.4	151.8	189.9	53.5	251.9	115.4	255.6	163.5
Hospital re-presentation within 30 days										
Outpatient	15.1	4.2	14.4	3.9	15.2	4.0	14.5	4.0	14.8	4.1
Inpatient	221.0	87.4	248.8	133.4	200.3	66.2	219.3	80.3	230.0	105.5

SD, Standard Deviation.

Inpatient costs increased with disease severity. The cost ranged from US$194.2 (SD 34.8) for episodes categorised as low severity to US$387.2 (SD 358.2) for episodes categorised as high severity. Costs also increased with the duration of stay (table 4). Corresponding with the increased length of stay for patients aged 0–11 months and older than 65 years, the mean costs were higher compared with other age categories (online supplemental table 8). The cost of malaria episodes per person-year of observation following an initial episode of *P. falciparum* (US$26.35) and *P. vivax* (US$26.50) was similar (online supplemental table 5).

Additional costs incurred for patients who presented again to the hospital for any reason within 30 days following their malaria episode. These costs were US$230.0 (SD 105.5) for patients requiring inpatient admission and US$14.8 (SD 4.1) for outpatient re-presentations (table 4). Non-malaria re-presentation as outpatients at the hospital incurred a cost of US$12.5 (SD 1.9) compared with a cost of US$17.4 (SD 4.3) for patients presenting again with malaria (online supplemental table 10). The costs for inpatient care at the hospital for non-malaria and for malaria re-presentations were US$284.9 (SD 190.0) and US$213.7 (SD 52.5), respectively (online supplemental table 10). Costs for inpatient re-presentations were higher for patients initially presenting with vivax malaria (US$248.8, SD 133.4) compared with those with falciparum malaria (US$221.0, SD 87.4); p=0.053 (table 4).

## Discussion

This study describes the distribution and presentation of malaria cases at healthcare facilities in Indonesia, including their discharge status, mortality and the costs related to hospitalisation, by leveraging the use of a 1% NHI claim data sample. While there were differences across provinces, *P. falciparum* episodes were more frequent than *P. vivax* overall. More than half of malaria cases were initially treated in the primary care facility, and only 3% of these patients were referred to the hospital. The majority of inpatient admissions were categorised as low severity, without any comorbidity and complication. On average, the malaria inpatient costs were 10-fold higher than the outpatient costs at the hospital level. Furthermore, we found that the costs of re-presentation to hospitals were higher in *P. vivax* patients compared with patients with *P. falciparum*.

The 1% sampled dataset used for this analysis includes 12 970 malaria episodes across 6 years, from 2015 to 2020, extrapolating to an estimated 1 297 000 cases that would have occurred across the entire population over this period. This figure corresponds well with the 1 423 876 malaria cases reported by the Ministry of Health’s routine electronic malaria information system (e-sysmal) during the same period in Indonesia.^4^ Our results also concurred with Ministry of Health data in terms of the distribution of cases by age, gender and geographical location.^4^ The WHO’s World Malaria Report indicates case numbers for Indonesia are up to twofold higher.^2^ This discrepancy is a likely reflection of WHO’s use of statistical projection for case estimation, compared with the surveillance data that capture the actual cases of patients who visited healthcare facilities.

Our analysis identified that 19% (2384/12 970) of malaria episodes were reported as ‘unspecified malaria’, coded by ICD-10 B54. ‘Unspecified malaria’ refers to malaria with clinical symptoms but without parasitological confirmation due to the limitation of diagnostic modalities or technical challenges in remote areas.^19^ The Ministry of Health data report the proportion of ‘suspect or clinical malaria diagnosis’ at 13% of the total malaria cases in 2022.^4^ In the NIH dataset, the proportion of this category has decreased over the years, indicating improvement in the availability of malaria diagnosis over time, from 21% in 2015 to 16% in 2020.

The acute costs of inpatient care were related to the severity of disease, the number of additional non-malaria diagnoses and the duration of stay. To our knowledge, this is the first time that it has been estimated for Indonesia. This analysis showed that the costs of both hospital outpatient care in 2020 (US$16.2) and inpatient care (US$228.7) were much higher than previously reported using WHO-CHOICE data, which estimated US$4 for primary care visits and US$69 for inpatient stays in 2017.^20 21^ WHO-CHOICE data include the list of estimated treatment costs by countries, using a health sector perspective that covers overhead costs such as programme and training costs as well as patient-level service delivery costs. Our results show that the WHO-CHOICE cost data collected in 2007–2008 are likely to be vastly outdated.^20^

Our analysis highlighted that almost 5% of patients presented again for further healthcare within 30 days of an initial episode of acute malaria; this included both outpatient (3.1%) and inpatient (1.7%) care. These estimates are consistent with the risk of adverse clinical outcomes following treatment of *P. vivax* in Papua, Indonesia requiring inpatient care within 30 days.^22^ Our analysis also illustrated that more than half of these re-presentations to the hospital were due to additional malaria episodes, which emphasises the importance of providing highly effective treatment and patients completing a full course of treatment to reduce those visits. Importantly, the indirect costs due to non-malarial illness requiring outpatient and inpatient care were US$12.5 and US$284.9, respectively.

While our mortality estimates were similar to longitudinal studies of patients presenting to the hospital in Papua in 2009 (0.32% and 0.23%, respectively for *P. falciparum* and *P. vivax*),^23^ a 10-fold difference was seen between our data and that reported by the Ministry of Health. While the overall malaria mortality estimated by the Ministry of Health was 0.04% (513/1 423 876) during 2015–2020,^4^ the mortality from the NHI dataset during the same period was 0.42% (54/12 970). This discrepancy may be explained by differences in the data coverage and sampling framework with the NHI data that could potentially result in the inclusion of more severe patients. Furthermore, the Ministry of Health data may be missing records from private facilities. Importantly, the NHI data record mortality for any patients with the malaria ICD-10 code, even if the cause of death was not related to malaria, which may have resulted in the overestimated risk of mortality.

Our analysis includes all service records in both public and private healthcare facilities that have been contracted by the *Badan Penyelenggara Jaminan Sosial (BPJS) Kesehatan*, which is the administering body of NHI and are available in the public domain. Critically, this large dataset includes private facilities that may be missing from the Ministry of Health data. Given that the coverage of the NHI scheme almost reaches the entire population in Indonesia, the service record of this dataset can be leveraged for priority diseases, including malaria. This can potentially bridge gaps in the routine electronic malaria information system managed by the Ministry of Health. Furthermore, the cost findings by the status of morbidity, severity level, types of services (inpatient or outpatient) and re-admission are available in this study, which can be a reference for further malaria economic evaluation studies.

Our study has several limitations. First, the costs related to treatment at primary care facilities could not be assessed. The NHI scheme pays primary care facilities by a capitation payment mechanism through which they receive a set amount of money to cover the predicted cost of all or some of the healthcare services for each patient registered at that facility each month, regardless of the number of visits.^24^ Second, inherent to the dataset used, there is no specific information on how malaria species were diagnosed or what treatment was prescribed. Therefore, this does not allow for an analysis of treatment outcomes by medication administered. Third, the dataset included only ICD-10 data, and not the most recent ICD-11 data. Fourth, only 83% of the total population is covered by the NHI, and the rest is uninsured and, therefore, not included in our analysis.^25^ This uninsured group may include vulnerable populations, such as those in remote areas or with lower socioeconomic status—characteristics common among patients with malaria. In addition, our dataset only includes patients who seek care in the public health system. Studies from Papua and Java suggest that only about half of patients with symptoms of fever seek care at public facilities.^26 27^ Lastly, Indonesia has been divided into 38 provinces since 2022, and our data use the old administrative division into 34 provinces.

## Conclusion

Integrating the NHI dataset into routine surveillance data could provide more granular data for the Ministry of Health to direct malaria control and elimination efforts. Notably, under the current regulations, the Ministry of Health would be able to access the complete NHI service record of the entire population in Indonesia, providing insights beyond the 1% sample analysed in this study.^22^ The NHI dataset includes data that are currently not collected through the Ministry of Health data capture system, including costs of treatment and referral patterns. Future studies should consider whether and how the routine malaria information system and the NHI dataset could be integrated, including the regulatory arrangement that would be needed to make this possible. There are opportunities to use similar approaches for other disease programmes.

## Supplementary material

10.1136/bmjgh-2024-018255 online supplemental file 1

10.1136/bmjgh-2024-018255 online supplemental figure 1

## Data Availability

Data are available upon reasonable request.
