# Supplementary material for: Malaria morbidity, mortality and associated costs in Indonesia: analysis of the National Health Insurance claim dataset
Source: BMJ Glob Health. 2025 May 12;10(5):e018255. doi: 10.1136/bmjgh-2024-018255 (PMC12083301; doi:10.1136/bmjgh-2024-018255)
Supplement: online supplemental file 1 [file bmjgh-10-5-s001.docx]

Table of contents

[Supplementary Table 1**.** Variables in the four National Health Insurance datasets. 2](#_Toc191819840)

[Supplementary Table 2. Case distribution reported in the National Health Insurance dataset by malaria species from 2015 to 2020. 3](#_Toc191819841)

[Supplementary Table 3. Distribution of malaria species in each province from the National Health Insurance sampled datasets 2015-2020. 4](#_Toc191819842)

[Supplementary Table 4. Number of patients with single or multiple episodes of malaria (N = 8,833). 5](#_Toc191819843)

[Supplementary Table 5. Number of malaria episodes, person-years of observation, and costs per person-years of observation started from the first malaria episode in the dataset, distributed by types of malaria. Costs are in 2020 United States Dollars 6](#_Toc191819844)

[Supplementary Table 6. Number of hospital episodes with additional non-malaria diagnosis by species 7](#_Toc191819845)

[Supplementary Table 7. Hospital presentation within 30 days after the first episodes of malaria 7](#_Toc191819846)

[Supplementary Table 9. The discharge status of malaria cases both in the primary care and hospital settings by types of malaria species (N=12,970). 10](#_Toc191819847)

[Supplementary Table 10. The costs of re-presentation to the hospital within 30 days after the first episode of malaria and disaggregated by event of malaria and non-malaria re-presentation. 11](#_Toc191819848)

[Supplementary Figure 1. Healthcare pattern of malaria episodes at primary care facilities and hospitals, including discharge status. (a) for any malaria; (b) for *P. falciparum;* and (c) for *P. vivax*. 12](#_Toc191819849)

# Supplementary Table 1**.** Variables in the four National Health Insurance datasets.

| Available variables | Membership | Primary care | Hospital | Secondary diagnosis |
| --- | --- | --- | --- | --- |
| Patient ID | ☑️ | ☑️ | ☑️ | ☑️ |
| Date of birth | ☑️ |  |  |  |
| Gender | ☑️ |  |  |  |
| Location of residence | ☑️ |  |  |  |
| Type of membership | ☑️ |  |  |  |
| Visits ID |  | ☑️ | ☑️ |  |
| Date of admission |  | ☑️ | ☑️ |  |
| Date of discharge |  | ☑️ | ☑️ |  |
| Discharge status  (i.e., referral) |  | ☑️ | ☑️ |  |
| Location of health facilities |  | ☑️ | ☑️ |  |
| Primary diagnosis code  (ICD-10) |  | ☑️ | ☑️ |  |
| Secondary diagnosis code |  |  |  | ☑️ |
| Procedure code |  |  | ☑️ |  |
| Type of facilities (public/private) |  | ☑️ | ☑️ |  |
| Type of services (outpatient/inpatient) |  | ☑️ | ☑️ |  |
| Type of department (Emergency/general ward) |  | ☑️ | ☑️ |  |
| Reimbursement rate |  |  | ☑️ |  |

# Supplementary Table 2. Case distribution reported in the National Health Insurance dataset by malaria species from 2015 to 2020.

| Year | *P. falciparum* | | *P. vivax* | | Other malaria | | Unspecified malaria | | Total |
| --- | --- | --- | --- | --- | --- | --- | --- | --- | --- |
|  | **n** | **%** | **n** | **%** | **n** | **%** | **n** | **%** | **n** |
| 2015 | 1,021 | 38.9% | 1,013 | 38.6% | 47 | 1.8% | 543 | 20.7% | 2,624 |
| 2016 | 1,440 | 48.3% | 977 | 32.8% | 70 | 2.3% | 496 | 16.6% | 2,983 |
| 2017 | 1,272 | 47.2% | 895 | 33.2% | 56 | 2.1% | 474 | 17.5% | 2,697 |
| 2018 | 812 | 45.9% | 559 | 31.6% | 38 | 2.2% | 359 | 20.3% | 1,768 |
| 2019 | 769 | 50.9% | 417 | 27.6% | 32 | 2.1% | 292 | 19.4% | 1,510 |
| 2020 | 705 | 50.8% | 446 | 32.1% | 17 | 1.2% | 220 | 15.9% | 1,388 |
| Total | **6,019** | **46.4%** | **4,307** | **33.2%** | **260** | **2.0%** | **2,384** | **18.4%** | **12,970** |

# Supplementary Table 3. Distribution of malaria species in each province from the National Health Insurance sampled datasets 2015-2020.

| Province* | *P. falciparum* | | *P. vivax* | | Other malaria | | Unspecified Malaria | | Total | |
| --- | --- | --- | --- | --- | --- | --- | --- | --- | --- | --- |
|  | n | % | n | % | n | % | n | % | n | %^†^ |
| Aceh | 18 | 58.1% | 4 | 12.9% | 2 | 6.5% | 7 | 22.6% | 31 | 0.2% |
| North Sumatera | 369 | 60.3% | 74 | 12.1% | 15 | 2.5% | 154 | 25.2% | 612 | 4.7% |
| West Sumatera | 56 | 50.0% | 14 | 12.5% | 2 | 1.8% | 40 | 35.7% | 112 | 0.9% |
| Riau | 22 | 33.8% | 3 | 4.6% | 1 | 1.5% | 39 | 60.0% | 65 | 0.5% |
| Jambi | 17 | 9.3% | 40 | 21.9% | 4 | 2.2% | 122 | 66.7% | 183 | 1.4% |
| South Sumatera | 222 | 34.9% | 32 | 5.0% | 6 | 0.9% | 377 | 59.2% | 637 | 4.9% |
| Bengkulu | 17 | 2.7% | 355 | 57.4% | 11 | 1.8% | 236 | 38.1% | 619 | 4.8% |
| Lampung | 35 | 18.1% | 36 | 18.7% | 1 | 0.5% | 121 | 62.7% | 193 | 1.5% |
| Bangka Belitung Islands | 16 | 15.8% | 68 | 67.3% | 1 | 1.0% | 16 | 15.8% | 101 | 0.8% |
| Riau Islands | 10 | 33.3% | 4 | 13.3% | 2 | 6.7% | 14 | 46.7% | 30 | 0.2% |
| Jakarta | 12 | 33.3% | 13 | 36.1% | 1 | 2.8% | 10 | 27.8% | 36 | 0.3% |
| West Jawa | 70 | 30.8% | 76 | 33.5% | 8 | 3.5% | 73 | 32.2% | 227 | 1.8% |
| Central Jawa | 52 | 31.0% | 56 | 33.3% | 7 | 4.2% | 53 | 31.5% | 168 | 1.3% |
| Yogyakarta | 7 | 25.0% | 10 | 35.7% | 0 | 0.0% | 11 | 39.3% | 28 | 0.2% |
| East Jawa | 58 | 38.2% | 48 | 31.6% | 8 | 5.3% | 38 | 25.0% | 152 | 1.2% |
| Banten | 1 | 5.3% | 8 | 42.1% | 1 | 5.3% | 9 | 47.4% | 19 | 0.1% |
| Bali | 7 | 33.3% | 2 | 9.5% | 1 | 4.8% | 11 | 52.4% | 21 | 0.2% |
| West Nusa Tenggara | 45 | 51.7% | 17 | 19.5% | 2 | 2.3% | 23 | 26.4% | 87 | 0.7% |
| East Nusa Tenggara | 500 | 41.7% | 551 | 46.0% | 30 | 2.5% | 118 | 9.8% | 1,199 | 9.2% |
| West Kalimantan | 17 | 33.3% | 4 | 7.8% | 1 | 2.0% | 29 | 56.9% | 51 | 0.4% |
| Central Kalimantan | 10 | 45.5% | 7 | 31.8% | 1 | 4.5% | 4 | 18.2% | 22 | 0.2% |
| South Kalimantan | 27 | 39.1% | 22 | 31.9% | 4 | 5.8% | 16 | 23.2% | 69 | 0.5% |
| East Kalimantan | 56 | 38.6% | 37 | 25.5% | 5 | 3.4% | 47 | 32.4% | 145 | 1.1% |
| North Kalimantan | 5 | 41.7% | 1 | 8.3% | 0 | 0.0% | 6 | 50.0% | 12 | 0.1% |
| North Sulawesi Utara | 103 | 47.2% | 70 | 32.1% | 7 | 3.2% | 38 | 17.4% | 218 | 1.7% |
| Central Sulawesi | 48 | 36.4% | 26 | 19.7% | 5 | 3.8% | 53 | 40.2% | 132 | 1.0% |
| South Sulawesi | 63 | 30.3% | 67 | 32.2% | 5 | 2.4% | 73 | 35.1% | 208 | 1.6% |
| Southeast Sulawesi | 24 | 26.4% | 33 | 36.3% | 5 | 5.5% | 29 | 31.9% | 91 | 0.7% |
| Gorontalo | 11 | 33.3% | 4 | 12.1% | 1 | 3.0% | 17 | 51.5% | 33 | 0.3% |
| West Sulawesi Barat | 4 | 21.1% | 7 | 36.8% | 0 | 0.0% | 8 | 42.1% | 19 | 0.1% |
| Maluku | 109 | 23.2% | 227 | 48.5% | 5 | 1.1% | 128 | 27.3% | 469 | 3.6% |
| North Maluku | 162 | 49.2% | 66 | 20.1% | 12 | 3.6% | 89 | 27.1% | 329 | 2.5% |
| West Papua | 452 | 41.4% | 528 | 48.4% | 18 | 1.6% | 93 | 8.5% | 1,091 | 8.4% |
| Papua | 3,394 | 61.0% | 1,797 | 32.3% | 88 | 1.6% | 282 | 5.1% | 5,561 | 42.9% |
| Total | **6,019** | **46.4%** | **4,307** | **33.2%** | **260** | **2.0%** | **2,384** | **18.4%** | **12,970** | **100%** |

*Order from the western to the eastern provinces of Indonesia.

**^†^**Percentage of column

# Supplementary Table 4. Number of patients with single or multiple episodes of malaria (N = 8,833).

| Number of patients | n | % |
| --- | --- | --- |
| Single episode | **6,676** | **75.6%** |
| *P. falciparum* | 2,934 | 33.2% |
| *P. vivax* | 2,141 | 24.2% |
| Other malaria | 1,601 | 18.1% |
| Two episodes | **1,330** | **15.1%** |
| *P. falciparum* after *P. falciparum* | 440 | 5.0% |
| *P. vivax* after *P. vivax* | 310 | 3.5% |
| *P. falciparum* after *P. vivax* | 133 | 1.5% |
| *P. vivax* after *P. falciparum* | 139 | 1.6% |
| Any other combination | 308 | 3.5% |
| More than two episodes | **827** | **9.4%** |
| All *P. falciparum* | 299 | 3.4% |
| All *P. vivax* | 195 | 2.2% |
| Any other combination | 333 | 3.8% |

Supplementary Table 5. Number of malaria episodes, person-years of observation, and costs per person-years of observation started from the first malaria episode in the dataset, distributed by types of malaria. Costs are in 2020 United States Dollars.

|  | *P. falciparum* | *P. vivax* | Other malaria | Unspecified malaria |
| --- | --- | --- | --- | --- |
| Total number of episodes* | **5,505** | **3,794** | **149** | **1,587** |
| Person years of observation | **14,705** | **11,470** | **595** | **4,959** |
| Malaria episodes per person-year of observation overall | **0.38** | **0.33** | **0.25** | **0.32** |
| 0-11 months | 0.27 | 0.26 | 0.25 | 0.38 |
| 1-5 years | 0.38 | 0.31 | 0.24 | 0.35 |
| 6-10 years | 0.35 | 0.34 | 0.25 | 0.28 |
| 11-15 years | 0.36 | 0.31 | 0.31 | 0.29 |
| 16-65 years | 0.37 | 0.33 | 0.27 | 0.32 |
| Over 65 years | 0.35 | 0.28 | 0.46 | 0.34 |
| Total hospital costs during the years of observation in the sampled dataset | **384,268** | **301,716** | **6,286** | **54,307** |
| Cost per person year of observation | **26.35** | **26.50** | **10.57** | **10.95** |

* Malaria episodes occurred from the first malaria episode in the dataset until December 2020.

Supplementary Table 6. Number of hospital episodes with additional non-malaria diagnosis by species.

|  | *P. falciparum* (n=3100) | | *P. vivax*  (n=2,150) | | Other malaria  (n=70) | | Unspecified malaria (n=644) | | Total  (N=5,964) | |
| --- | --- | --- | --- | --- | --- | --- | --- | --- | --- | --- |
|  | **n** | **%** | **n** | **%** | **n** | **%** | **n** | **%** | **n** | **%** |
| Only malaria diagnosis | 1,488 | 48.0% | 956 | 44.0% | 34 | 5% | 244 | 38% | 2,722 | 45.6% |
| Malaria plus one additional diagnosis | 868 | 28.0% | 706 | 33.0% | 21 | 3% | 242 | 38% | 1,837 | 30.8% |
| Malaria plus two additional diagnoses | 460 | 15.0% | 312 | 15.0% | 5 | 1% | 116 | 18% | 893 | 14.9% |
| Malaria plus more than two additional diagnoses | 284 | 9.0% | 176 | 8.0% | 10 | 2% | 42 | 7% | 512 | 8.6% |

# Supplementary Table 7. Hospital presentation within 30 days after the first episodes of malaria

|  | *P. falciparum* (N=6,019) | | *P. vivax*  (N=4,307) | | Other malaria  (N=260) | | Unspecified malaria (N=2,384) | | Total  (N=12,970) | |
| --- | --- | --- | --- | --- | --- | --- | --- | --- | --- | --- |
|  | **n** | **%** | **n** | **%** | **n** | **%** | **n** | **%** | **n** | **%** |
| No repeated hospital visit within 30 days | **5,684** | **94.4%** | **4,128** | **95.8%** | **250** | **96.1%** | **2,285** | **95.8%** | **12,347** | **95.1%** |
| Hospital presentation within 30 days as outpatient | **213** | **3.5%** | **102** | **2.4%** | **3** | **1.2%** | **86** | **3.6%** | **404** | **3.1%** |
| Non-malaria first diagnosis | 105 | 49.3% | 44 | 43.1% | 2 | 66.7% | 61 | 70.9% | 212 | 52.5% |
| Malaria first diagnosis | 108 | 50.7% | 58 | 56.9% | 1 | 33.3% | 25 | 29.1% | 192 | 47.5% |
| Hospital presentation within 30 days as inpatient | **122** | **2.1%** | **77** | **1.8%** | **7** | **2.7%** | **13** | **0.5%** | **219** | **1.7%** |
| Non-malaria first diagnosis | 27 | 22.1% | 17 | 22.1% | 1 | 14.3% | 5 | 38.5% | 50 | 22.8% |
| Malaria first diagnosis | 95 | 77.9% | 60 | 77.9% | 6 | 85.7% | 8 | 61.5% | 169 | 77.2% |

Supplementary Table 8. Age-stratified hospital presentations, costs, and length of stay for malaria treatment by species. Costs are in 2020 United States dollars.

|  | *P. falciparum* (N=3,100) | | *P. vivax*  (N=2,150) | | Other malaria  (N=70) | | Unspecified malaria (N=644) | | Total  (N=5,964) | |
| --- | --- | --- | --- | --- | --- | --- | --- | --- | --- | --- |
| All hospital presentations* | **n** | **%** | **n** | **%** | **n** | **%** | **n** | **%** | **n** | **%** |
| All ages | 3,100 | 100% | 2,150 | 100% | 70 | 100% | 644 | 0.3% | 5964 | 100% |
| 0-11 months | 39 | 1.3% | 38 | 1.8% | 2 | 2.9% | 7 | 1.7% | 86 | 100% |
| 1-5 years | 203 | 6.5% | 198 | 9.2% | 2 | 2.9% | 37 | 1.8% | 440 | 100% |
| 5-10 years | 261 | 8.4% | 206 | 9.6% | 4 | 5.7% | 39 | 1.4% | 510 | 100% |
| 10-15 years | 258 | 8.3% | 141 | 6.6% | 3 | 4.3% | 31 | 23.9% | 433 | 100% |
| 15-65 years | 2,214 | 71.4% | 1,493 | 69.4% | 59 | 84.3% | 513 | 0.8% | 4279 | 100% |
| Over 65 years | 125 | 4.0% | 74 | 3.4% | 0 | 0.0% | 17 | 0.3% | 216 | 100% |
| Hospital outpatient costs | **Mean** | **SD^†^** | **Mean** | **SD^†^** | **Mean** | **SD^†^** | **Mean** | **SD^†^** | **Mean** | **SD^†^** |
| All ages | 16.6 | 4.1 | 15.9 | 4.3 | 16.5 | 3.2 | 15.5 | 4.1 | 16.2 | 3.6 |
| 0-11 months | 16.1 | 4.4 | 13.8 | 3.9 | 12.1 | 1.4 | 16.0 | 4.7 | 14.9 | 4.5 |
| 1-5 years | 14.6 | 3.7 | 14.3 | 3.9 | N/A | N/A | 14.4 | 3.9 | 14.4 | 3.2 |
| 5-10 years | 15.2 | 4.3 | 15.3 | 4.3 | N/A | N/A | 13.7 | 3.9 | 16.6 | 3.5 |
| 10-15 years | 16.4 | 4.3 | 16.8 | 4.3 | N/A | N/A | 17.6 | 4.5 | 16.6 | 3.5 |
| 15-65 years | 17.0 | 4.2 | 16.1 | 4.4 | 16.9 | 4.2 | 15.6 | 4.8 | 16.5 | 3.5 |
| Over 65 years | 15.9 | 4.0 | 15.4 | 5.3 | N/A | N/A | 14.6 | 4.8 | 15.1 | 3.3 |
| Length of inpatient stay (days) | **Mean** | **SD^†^** | **Mean** | **SD^†^** | **Mean** | **SD^†^** | **Mean** | **SD^†^** | **Mean** | **SD^†^** |
| All ages | 3.7 | 3.0 | 3.9 | 3.1 | 3.22 | 2.19 | 4.11 | 2.77 | 3.56 | 2.90 |
| 0-11 months | 5.2 | 4.6 | 3.9 | 2.1 | N/A | N/A | N/A | N/A | 4.48 | 3.40 |
| 1-5 years | 3.2 | 2.0 | 3.3 | 1.5 | 3.0 | 1.6 | 3.0 | 1.6 | 3.23 | 1.72 |
| 5-10 years | 3.9 | 3.5 | 3.4 | 2.8 | 3.3 | 0.7 | 3.3 | 0.7 | 3.32 | 2.68 |
| 10-15 years | 3.8 | 2.8 | 3.8 | 3.5 | 2.3 | 1.2 | 2.3 | 1.2 | 3.55 | 2.66 |
| 15-65 years | 3.6 | 2.7 | 3.9 | 3.2 | 3.4 | 2.4 | 3.4 | 2.4 | 3.56 | 2.86 |
| Over 65 years | 5.3 | 5.6 | 6.0 | 4.4 | N/A | N/A | N/A | N/A | 5.32 | 4.68 |
| Hospital inpatient costs (USD) | **Mean** | **SD^†^** | **Mean** | **SD^†^** | **Mean** | **SD^†^** | **Mean** | **SD^†^** | **Mean** | **SD^†^** |
| All ages | 129.5 | 129.5 | 99.4 | 119.1 | 202.1 | 51.1 | 236.6 | 104.2 | 228.7 | 122.6 |
| 0-11 months | 264.9 | 117.3 | 255.6 | 76.9 | N/A | N/A | 315 | 250 | 263.9 | 111.3 |
| 1-5 years | 219.5 | 58.4 | 230.5 | 64.7 | 264.1 | 115.4 | 264 | 159 | 227.9 | 71.2 |
| 5-10 years | 223.9 | 75.9 | 217.9 | 56.4 | 190.7 | 17.5 | 266 | 169 | 224.5 | 100.8 |
| 10-15 years | 234.8 | 122.0 | 211.9 | 59.7 | 198.4 | 54.5 | 206 | 66 | 224.5 | 100.8 |
| 15-65 | 222.1 | 138.2 | 232.1 | 130.4 | 200.3 | 49.7 | 229 | 89 | 226.4 | 129.9 |
| Over 65 years | 259.1 | 148.9 | 270.1 | 167.4 | N/A | N/A | 352 | 157 | 223.1 | 76.2 |
| Hospital inpatient costs (USD) | **Mean** | **SD^†^** | **Mean** | **SD^†^** | **Mean** | **SD^†^** | **Mean** | **SD^†^** | **Mean** | **SD^†^** |
| All inpatient class | 225.56 | 129.55 | 202.07 | 51.06 | 202.07 | 51.06 | 236.57 | 104.19 | 228.72 | 122.62 |
| Class I | 272.43 | 220.94 | 246.29 | 56.82 | 246.29 | 56.82 | 294.42 | 116.16 | 267.66 | 159.32 |
| Class II | 225.39 | 92.32 | 205.04 | 36.49 | 205.04 | 36.49 | 228.08 | 100.26 | 225.06 | 90.77 |
| Class III | 207.52 | 91.12 | 184.50 | 47.42 | 184.50 | 47.42 | 205.80 | 82.05 | 210.46 | 116.42 |

*all hospital presentation includes both inpatient and outpatient care at the hospital level

**^†^** SD = Standard deviation

# Supplementary Table 9. The discharge status of malaria cases both in the primary care and hospital settings by types of malaria species (N=12,970).

|  | *P. falciparum* | | *P. vivax* | | Other malaria | | Unspecified Malaria | | Total | |
| --- | --- | --- | --- | --- | --- | --- | --- | --- | --- | --- |
|  | n | % | n | % | n | % | n | % | n | % |
| Primary Health care | **2,919** | **48.5%** | **2,157** | **50.1%** | **190** | **73.1%** | **1,740** | **73.0%** | **7,006** | **54.0%** |
| Private General Practice | 842 | 28.8% | 804 | 37.3% | 44 | 23.2% | 482 | 27.7% | 2,172 | 31.0% |
| Treated as outpatient | 822 | 97.6% | 796 | 99.0% | 35 | 79.5% | 479 | 99.4% | 2,132 | 98.2% |
| Referred to hospital | 17 | 2.0% | 8 | 1.0% | 9 | 20.5% | 3 | 0.6% | 37 | 1.7% |
| No further health care encounters | 3 | 0.4% |  | 0.0% | - | 0.0% | 0 | 0.0% | 3 | 0.1% |
| Private Clinics | 608 | 20.8% | 603 | 28.0% | 26 | 13.7% | 501 | 28.8% | 1,738 | 24.8% |
| Treated as outpatient | 340 | 55.9% | 331 | 54.9% | 13 | 50.0% | 419 | 83.6% | 1,103 | 63.5% |
| Internal referral | 0 | 0.0% | 9 | 1.5% | - | 0.0% | 0 | 0.0% | 9 | 0.5% |
| Referred to hospital | 37 | 6.1% | 11 | 1.8% | 7 | 26.9% | 34 | 6.8% | 89 | 5.1% |
| No further health care encounters | 231 | 38.0% | 251 | 41.6% | 6 | 23.1% | 48 | 9.6% | 536 | 30.8% |
| Others | 0 | 0.0% | 1 | 0.2% | - | 0.0% | 0 | 0.0% | 1 | 0.1% |
| Puskesmas | 1,469 | 50.3% | 750 | 34.8% | 120 | 63.2% | 757 | 43.5% | 3,096 | 44.2% |
| Treated as outpatient | 896 | 61.0% | 501 | 66.8% | 68 | 56.7% | 608 | 80.3% | 2,073 | 67.0% |
| Refused further care | 2 | 0.1% | 1 | 0.1% | 1 | 0.8% | 0 | 0.0% | 4 | 0.1% |
| Internal referral | 0 | 0.0% | 0 | 0.0% | - | 0.0% | 2 | 0.3% | 2 | 0.1% |
| Referred to hospital | 37 | 2.5% | 14 | 1.9% | 8 | 6.7% | 22 | 2.9% | 81 | 2.6% |
| No further health care encounters | 526 | 35.8% | 231 | 30.8% | 42 | 35.0% | 125 | 16.5% | 924 | 29.8% |
| Others | 8 | 0.5% | 3 | 0.4% | 1 | 0.8% | 0 | 0.0% | 12 | 0.4% |
| Hospital | **3,100** | **51.5%** | **2,150** | **49.9%** | **70** | **26.9%** | **644** | **27.0%** | **5,964** | **46.0%** |
| Outpatient | 1,382 | 44.6% | 791 | 36.8% | 22 | 31.4% | 331 | 51.4% | 2,526 | 42.4% |
| Referred to other hospital | 1 | 0.1% | 0 | 0.0% | - | 0.0% | 3 | 0.9% | 4 | 0.2% |
| No further health care encounters | 1,380 | 99.9% | 789 | 99.7% | 22 | 100.0% | 327 | 98.8% | 2,518 | 99.7% |
| Inpatient | 1,718 | 55.4% | 1,359 | 63.2% | 48 | 68.6% | 313 | 48.6% | 3,438 | 57.6% |
| Dead | 36 | 2.1% | 16 | 1.2% | 1 | 2.1% | 1 | 0.3% | 54 | 1.6% |
| Refused further care | 48 | 2.8% | 42 | 3.1% | 2 | 4.2% | 6 | 1.9% | 98 | 2.9% |
| Referred to other hospital | 20 | 1.2% | 11 | 0.8% | 1 | 2.1% | 4 | 1.3% | 36 | 1.0% |
| No further health care encounters | 1,607 | 93.5% | 1,280 | 94.2% | 43 | 89.6% | 301 | 96.2% | 3,231 | 94.0% |
| Unknown | 7 | 0.4% | 10 | 0.7% | 1 | 2.1% | 1 | 0.3% | 19 | 0.6% |

# Supplementary Table 10. The costs of re-presentation to the hospital within 30 days after the first episode of malaria and disaggregated by event of malaria and non-malaria re-presentation.

|  | *P. falciparum* | | | *P. vivax* | | | Total | | |
| --- | --- | --- | --- | --- | --- | --- | --- | --- | --- |
|  | **n** | **Mean** | **SD*** | **n** | **Mean** | **SD*** | **n** | **Mean** | **SD*** |
| Hospital re-presentation for any reason (outpatient and inpatient) | **335** | **90.1** | **112.3** | **179** | **115.3** | **145.5** | **623** | **90.5** | **120.4** |
| Any outpatient re-presentation with malaria | 108 | 18.0 | 4.2 | 58 | 15.6 | 4.2 | 192 | 17.4 | 4.3 |
| Any outpatient re-presentation without malaria | 105 | 12.2 | 1.1 | 44 | 12.8 | 4.1 | 212 | 12.5 | 1.9 |
| Any inpatient re-presentation with malaria | 95 | 210.1 | 50.1 | 60 | 218.0 | 58.4 | 169 | 213.7 | 52.5 |
| Any inpatient re-presentation without malaria | 27 | 259.3 | 156.7 | 17 | 357.6 | 236.4 | 50 | 284.9 | 190.0 |

*SD = Standard deviation
