# Supplementary material for: Malaria morbidity, mortality and associated costs in Indonesia: analysis of the National Health Insurance claim dataset
Source: BMJ Glob Health. 2025 May 12;10(5):e018255. doi: 10.1136/bmjgh-2024-018255 (PMC12083301; doi:10.1136/bmjgh-2024-018255)
Supplement: online supplemental figure 1 [file bmjgh-10-5-s002.pdf]

a.

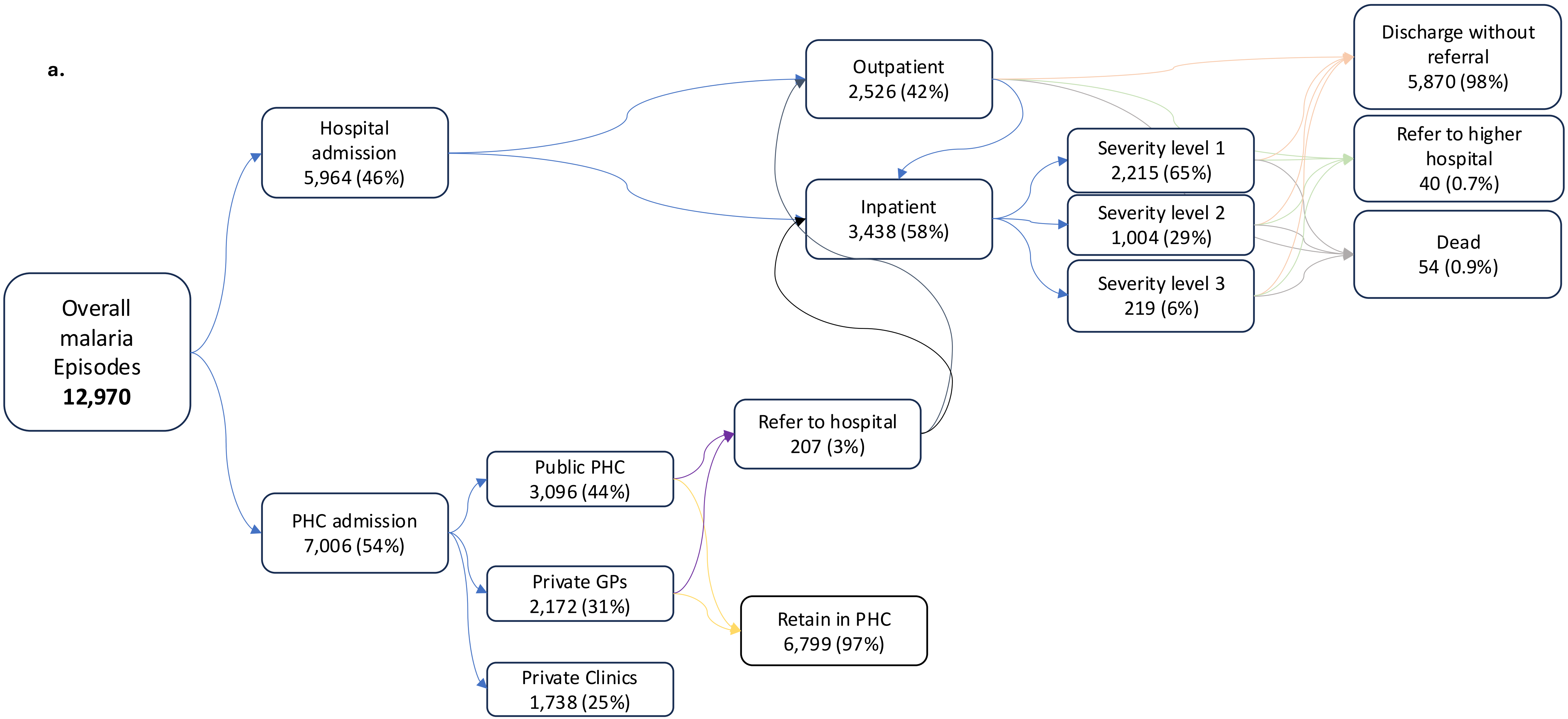
Overall malaria Episodes 12,970
Hospital admission 5,964 (46%)
PHC admission 7,006 (54%)
Outpatient 2,526 (42%)
Inpatient 3,438 (58%)
Severity level 1 2,215 (65%)
Severity level 2 1,004 (29%)
Severity level 3 219 (6%)
Discharge without referral 5,870 (98%)
Refer to higher hospital 40 (0.7%)
Dead 54 (0.9%)
Public PHC 3,096 (44%)
Private GPs 2,172 (31%)
Private Clinics 1,738 (25%)
Refer to hospital 207 (3%)
Retain in PHC 6,799 (97%)

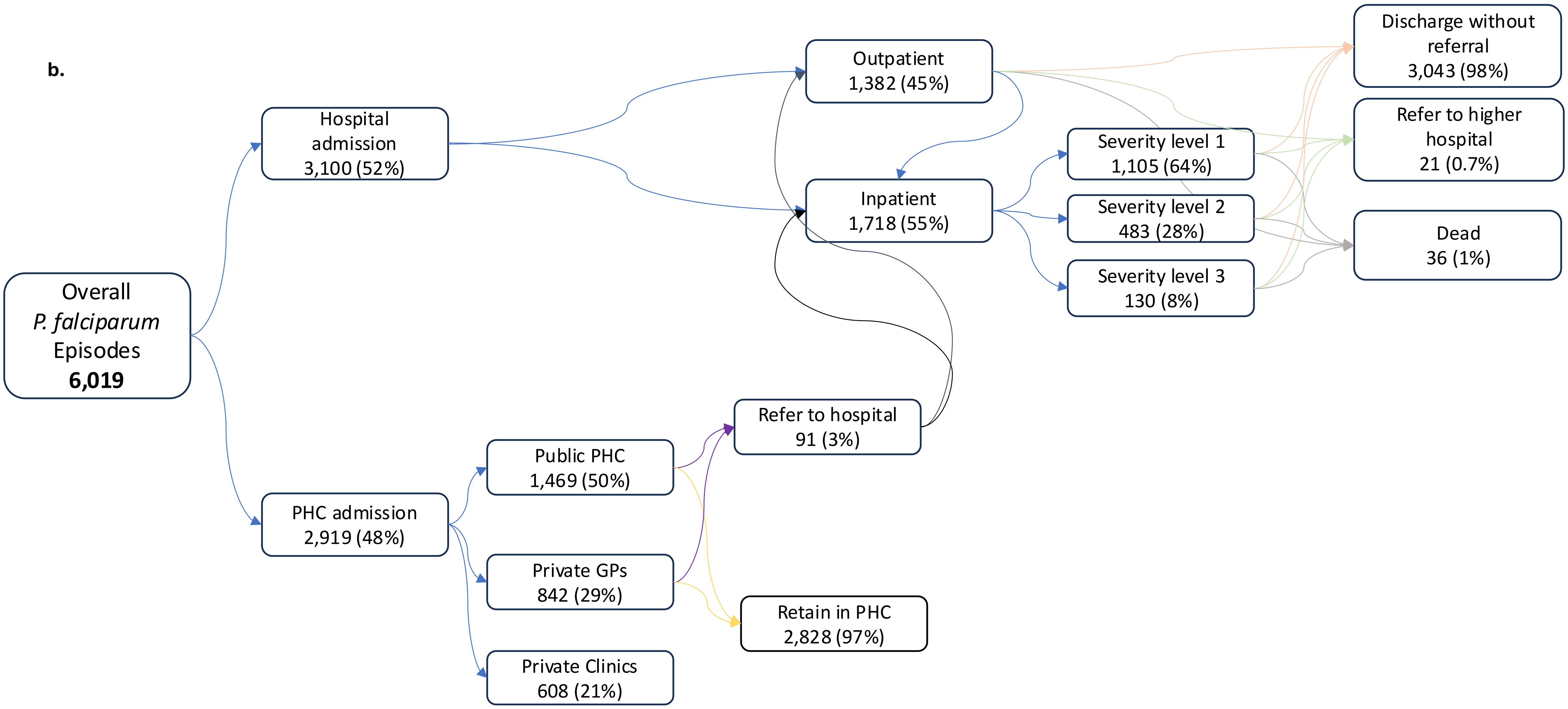

b.
Overall
P. falciparum
Episodes
6,019
Hospital admission
3,100 (52%)
PHC admission
2,919 (48%)
Outpatient
1,382 (45%)
Inpatient
1,718 (55%)
Severity level 1
1,105 (64%)
Severity level 2
483 (28%)
Severity level 3
130 (8%)
Discharge without referral
3,043 (98%)
Refer to higher hospital
21 (0.7%)
Dead
36 (1%)
Public PHC
1,469 (50%)
Private GPs
842 (29%)
Private Clinics
608 (21%)
Refer to hospital
91 (3%)
Retain in PHC
2,828 (97%)

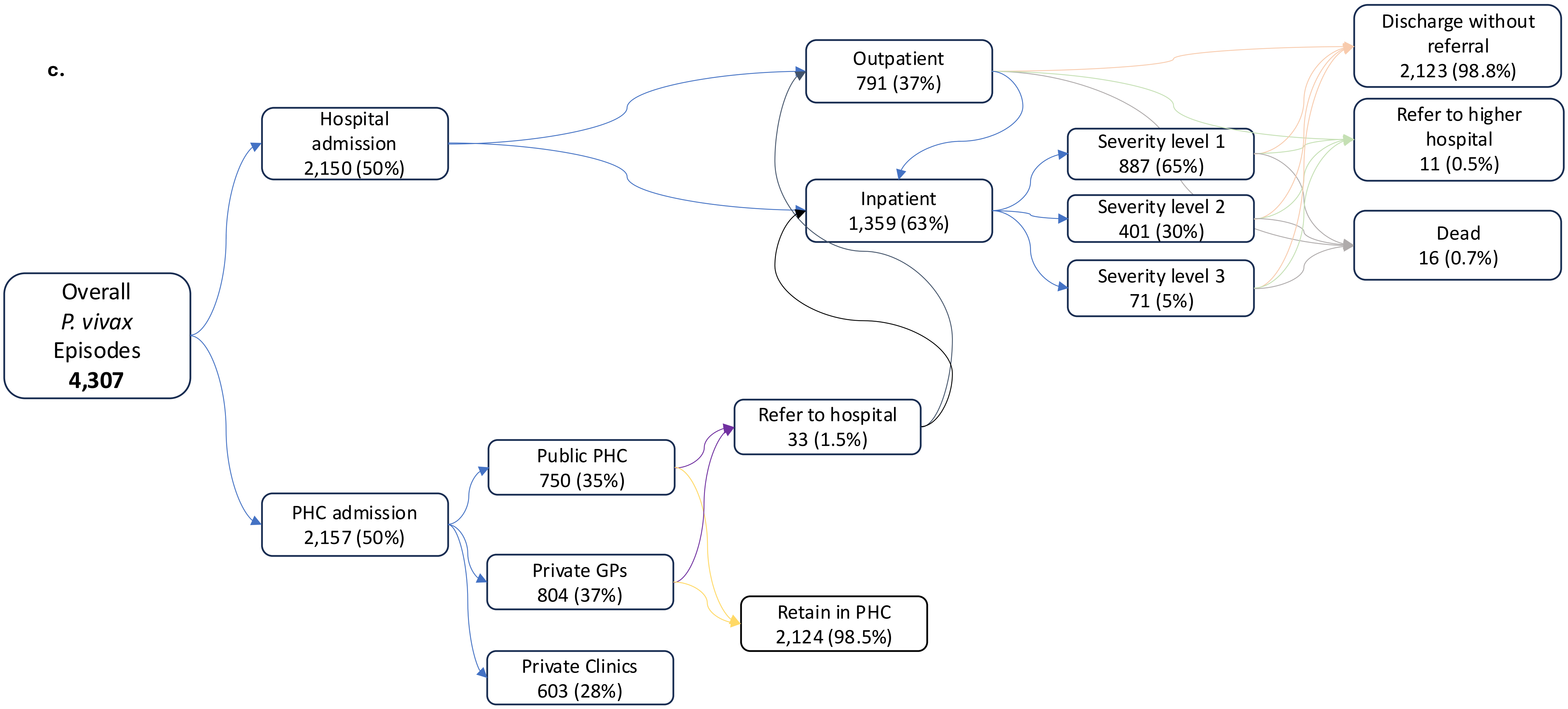

Supplementary Figure 1. Healthcare pattern of malaria episodes at primary care facilities and hospitals, including discharge status. (a) for any malaria; (b) for P. falciparum; and (c) for P. vivax.
